# Comparative study of the effect of National Health Insurance Scheme on use of delivery and antenatal care services between rural and urban women in Ghana

**DOI:** 10.1186/s13561-022-00357-z

**Published:** 2022-02-12

**Authors:** Raymond Elikplim Kofinti, Emmanuel Ekow Asmah, Edward Kwabena Ameyaw

**Affiliations:** 1 Department of Data Science and Economic Policy, School of Economics, University of Cape Coast, Cape Coast, Ghana; 2 The Australian Centre for Public and Population Health Research, Faculty of Health, University of Technology Sydney, Sydney, Australia

**Keywords:** NHIS, ANC, Delivery care, Rural-Ghana and urban-Ghana

## Abstract

**Background:**

Despite the focus of the National Health Insurance Scheme (NHIS) to bridge healthcare utilisation gap among women in Ghana, recent evidence indicates that most maternal deaths still occur from rural Ghana. The objective of this study was to examine the rural-urban differences in the effects of NHIS enrolment on delivery care utilisation (place of delivery and assistance at delivery) and antenatal care services among Ghanaian women.

**Methods:**

A nationally representative sample of 4169 women from the 2014 Ghana Demographic and Health Survey was used. Out of this sample, 2880 women are enrolled in the NHIS with 1229 and 1651 being urban and rural dwellers, respectively. Multivariate logistic and negative binomial models were fitted as the main estimation techniques. In addition, the Propensity Score Matching technique was used to verify rural-urban differences.

**Results:**

At the national level, enrolment in NHIS was observed to increase delivery care utilisation and the number of ANC visits in Ghana. However, rural-urban differences in effects were pronounced: whereas rural women who are enrolled in the NHIS were more likely to utilise delivery care [delivery in a health facility (OR = 1.870; CI = 1.533–2.281) and assisted delivery by a medical professional (OR = 1.994; CI = 1.631–2.438)], and have a higher number of ANC visits (IRR = 1.158; CI = 1.110–1.208) than their counterparts who are not enrolled, urban women who are enrolled in the NHIS on the other hand, recorded statistically insignificant results compared to their counterparts not enrolled. The PSM results corroborated the rural-urban differences in effects.

**Conclusion:**

The rural-urban differences in delivery and antenatal care utilisation are in favour of rural women enrolled in the NHIS. Given that poverty is endemic in rural Ghana, this positions the NHIS as a potential social equaliser in maternal health care utilisation especially in the context of developing countries by increasing access to delivery care services and the number of ANC visits.

## Background

Bridging the inequality gap in the access and utilisation of maternal health care services within countries has been a global concern, as echoed by the tenth Sustainable Development Goal (SDG) [1]. The disparity in maternal healthcare access and utilisation within countries constitutes one of the cardinal areas where inequality manifests strongly, and as a result, the SDG three seeks to offset in-country maternal healthcare inequalities and guarantee equal access to achieve less than 70 maternal mortality by the year 2030 [1]. These global efforts put Ghana at the forefront of maternal healthcare discourse. In Ghana, about four out of every five (79%) pregnant women delivered in a health facility and the same percentage received assistance from skilled medical professionals during childbirth [2]. Also, almost every pregnant woman (98%) in Ghana accesses and utilises ANC services in the country. Though these proportions are impressive, rural-urban differences call for concern as 90% urban women delivered and were assisted in a health facility compared to their rural counterparts of only 68% utilisation [3]. In most cases, rural women are at the disadvantaged end of inadequate access and utilisation [3–5] despite the introduction of the National Health Insurance Scheme (NHIS) in 2003 [6].

Though Maternal Mortality Ratio (MMR) declined by 49% in Ghana averaging 319 per 100,000 live births between 2011 and 2015, the rate is higher than the average for developing countries which stands at 239 per 100,000 live births [7]. Akin to observations at the global level [8], the highest proportion of maternal deaths in Ghana occur in resource-poor and rural locations [5]. This has been linked with the rural-urban disparity in maternal healthcare utilisation [3]. Beyond the direct causes of maternal mortality in Ghana such as haemorrhage, unsafe abortion and hypertensive conditions, the indirect factors seem to put women in rural Ghana at high risk. These include limited skilled health personnel, poverty, poor transport system, socio-cultural factors and insufficient blood at healthcare facilities [9, 10].

To offset all forms of disparities and eliminate out of pocket healthcare expenditure, the NHIS was established under the National Health Insurance Scheme (NHIS) Act 650, which was passed in 2003 and amended to ACT 852 in 2012. It envisages to “*ensure equitable and universal access for all residents of Ghana to an acceptable quality package of essential healthcare”* [6]. Under the pro-poor NHIS, antenatal care (ANC), delivery, postnatal care and free neonatal care for up to three months are comprehensively covered [11, 12]. Arguably, the pro-poor NHIS is equally pro-rural, considering the persistent dominance of poverty in rural Ghana compared to the urban locations [13, 14]. The incidence of poverty in rural Ghana (38.2%) is nearly four times higher compared with that of urban settings (10.4%) [15].

Despite the focus of the NHIS to bridge the equity gap within the country, recent evidence indicates that most maternal deaths still emanate from rural Ghana [5, 16]. This is entwined with the rural-urban disparity in maternal healthcare utilisation because according to the 2017 Maternal and Health Survey, 30% of all deliveries and stillbirths occurred at home among rural women compared with 9% for urban women. Moreover, 84% of urban women received all three maternal services while 64% of rural women had same [4]. Similar findings have been unravelled by some empirical studies [17, 18] while rural-urban disparity in healthcare quality has also been questioned [4].

The nexus between the NHIS and maternal healthcare utilisation in Ghana has received considerable attention in the literature. These include how the NHIS has facilitated maternal healthcare utilisation across wealth status of women [19], relationship between the NHIS and ANC visits [9, 10], effectiveness of the free maternal care under the NHIS [20, 21] as well as NHIS and general maternal healthcare access or utilisation [21–28]. The role of NHIS in maternal healthcare in Ghana has been compared with other countries as well [25]. The common ground of these studies is the acknowledgement that, the NHIS has been very instrumental in maternal healthcare utilisation, however, none of these studies is suggestive of the rural-urban differences in delivery and antenatal care utilisation vis-a-vis the NHIS.

This study examines the rural-urban differences in the effects of enrolment in NHIS on delivery care utilisation and the number of ANC visits to unravel the heterogeneity in maternal health care utilisation in the two-locations of the country. In our opinion, understanding the rural-urban contextual facilitating and inhibiting factors can help better appreciate whether the NHIS is serving the maternal healthcare needs of these categories of women equally or otherwise. The results will also provide the basis to ascertain whether there is the need to consider location-specific interventions and retooling of the NHIS to ensure that none of the Ghanaian women in their reproductive years is left behind in the utilisation of delivery care and ANC services in the country. The study thus contributes to the existing empirical studies on the association between NHIS and maternal health care utilisation with a specific focus on the rural-urban differences thereby contributing to the tenth SDG goal of offsetting within inequalities among Ghanaian women in the usage of delivery care and ANC.

We focus on testing women utilisation of delivery care services (Place of Delivery and Assistance at Birth); and their compliance to regular ANC visits (WHO’s standard of at least eight contacts with a health provider before delivery) vis-a-vis enrolment in the NHIS. In order to make attributions at the national level, three hypotheses were tested: (1) Pregnant women who are enrolled in the NHIS are more likely to deliver in a health facility compared to their counterparts who are not enrolled in the NHIS; (2) Pregnant women who are enrolled in the NHIS are more likely to be assisted by a medical professional during delivery in a health facility compared to their counterparts who are not enrolled in the NHIS; (3) The occurrence of regular ANC visits are higher for pregnant women who are enrolled in the NHIS compared to their counterparts who are not enrolled on to the Scheme. In addition, the evidence of rural-urban differences in attributions was garnered from three corresponding hypotheses: (4) Pregnant-rural women enrolled in the NHIS are more likely to deliver in a health facility compared to pregnant-urban women enrolled in the NHIS; (5) Pregnant-rural women enrolled in the NHIS are more likely to use the assistance of a medical professional during delivery compared to pregnant-urban women enrolled in the NHIS; and (6) The occurrence of regular ANC visits are higher for pregnant rural women enrolled in the NHIS compared to pregnant urban women enrolled in the scheme.

The broad framework for the current study is the Andersen health behaviour model which is a conventional tool for studies on health service utilisation [29]. The model postulates that health service utilisation is a function of three sets of factors, namely predisposing, enabling and need factors [30]. Whereas the predisposing and need factors are valid predictors of health service utilisation, our study is fashioned from the perspective of a key enabling factor focusing on NHIS subscription among women in their reproductive years and maternal healthcare utilisation. By this framework, we conjecture that Ghanaian women who are enrolled into the NHIS will be ‘enabled’ to utilise ANC and delivery care services compared to their counterparts who are not enabled. Unlike [31] who adjudged that enabling factors such as health insurance could engender inequity, our study conjecture that NHIS will rather bridge the inequality between rural and urban maternal healthcare utilisation.

## Materials and methods

### Data source

We deployed the 2014 Ghana Demographic and Health Survey (GDHS) which is a nationally representative survey administered by the Ghana Statistical Service (GSS). The 2014 GDHS employed a two-staged stratified sample frame where systematic sampling with probability proportional to size was used to identify enumeration areas from which households were selected based on 2010 Population and Housing Census.

The GDHS covered 9396 eligible women aged 15–49 out of 9656 registering a response rate of 97.3%. Our focus group was women with birth histories within the past five years preceding the survey. This group constitutes 4294 women. However, after managing the data and accounting for missing observations across the three dependent variables and twelve independent variables in the inferential analyses, our total comparable sample size reduced to 4169 registering an attrition rate of 2.9%. The rural and urban sub-samples considered for the analyses are 2457 and 1712 women respectively. It is worth mentioning that the nonproportional allocation of the women sample to different regions and to their urban and rural areas using the GDHS can cause differences in probability of selection and response rates in our sample distribution. The study adjusted for these concerns by applying individual weight for women using analytic weight for the descriptive statistics and by declaring our survey design to include the individual weight variable for women divided by 1,000,000 in the case of the inferential analyses.

### Definition of variables

#### Dependent variable

Three main variables were used to measure delivery and antenatal care utilisation, namely place of delivery, assistance at delivery and the number of ANC visits. The place of delivery is a binary dependent variable which measures whether the delivery took place at a health facility or otherwise. Deliveries that took place at a health facility were recoded as one (1), otherwise zero (0). Assistance at delivery measures whether the birth attendant is a trained medical professional or otherwise. Birth attendants in the categories of doctor, nurse, midwife and community health officers were recoded as one (1), otherwise zero. The number of ANC visits, on the other hand, is a count variable measuring the number of antenatal care visits made during pregnancy. Whereas the first two dependent variables depend on the availability of health facilities and skilled medical professionals, the third depends on the medical condition and needs of the specific woman. However, with WHO’s current recommended number of visits of at least eight (8) as of December 2017, regular visits are encouraged for expectant mothers, than otherwise.

#### Independent variables

The leading independent variable is enrolment in Ghana’s NHIS program. Respondents who are enrolled in the scheme were coded as one, and zero for those who are not enrolled.

The study also controlled for demographic, socio-economic and locational factors that influence maternal health care utilisation. The demographic variables include age which was measured as current age in completed years, marital status recoded as (1 = never married; 2 = currently married; 3 = Formerly married), ethnicity dummies (1 = Akan; 2 = Ga; 3 = Ewe; 4 = Northern), and religion dummies (1 = Christian; 2 = Moslem; 3 = Traditional; 4 = No religion). The socio-economic variables include mothers’ level of education recoded (1 = no level of schooling; 2 = primary education; 3 = secondary school and beyond), employment status of mothers was recoded (0 = not employed; 1 = employed), wealth quintile which is a composite index constructed from household asset data and dwelling characteristics using principal component analyses was coded as (1 = poorest; 2 = poorer; 3 = middle; 4 = rich/richest).

Two locational factors were used in the analyses, namely residential dummy (0 = rural; 1 = urban) and regional dummies (1 = Western; 2 = Central; 3 = Greater Accra; 4 = Volta; 5 = Eastern; 6 = Ashanti; 7 = Brong Ahafo; 8 = Northern; 9 = Upper East; 10 = Upper West). The PCA was used to create a continuous variable from barriers to seeking medical care (getting permission to go for treatment; getting the money needed for treatment; distance to health facility; not wanting to go alone). Each of the mentioned variables was recoded as one (1) in the case of a big problem, and zero (0) otherwise. Hence the PCA is imposed on these dummies to derive a continuous variable representing the barriers to medical care with Kaiser-Meyer-Olkin measure (KMO) of 0.65.

### Econometric analyses

The study deployed two main estimation techniques, the binary logistic and the negative binomial estimation techniques. The choice of the two variant estimation techniques was underscored by the six hypotheses of the study, measurement of the dependent variables and the need to correct for biases associated with overdispersion in the data. To suggest attributions, the results from the mentioned estimation techniques were verified using a quasi-experimental approach in the Propensity Score Matching. Subsequent sub-sections provide a brief description of the analytical tools deployed.

### Binary logistic estimation technique

The odds ratio variant of the logistic estimation technique was used to examine the rural-urban effects of NHIS enrolment on the place of delivery and the delivery care provided. This is because the two dependent variables are binary outcomes variables. The two models are specified as:

1 $$ \mathit{\ln}\left(\frac{\lambda_i}{1-{\lambda}_i\ }\right)={\beta}_0+{\beta}_1{NHIS}_i+{\beta}_2{WQ}_i+{\beta}_3{EDUC}_i+{\beta}_4{E\mathrm{M}P}_i+{\beta}_5\ {MAR}_i+{\beta}_6{AGE}_i+{\beta}_7\kern0.50em {REL}_i+{\beta}_8{ETHN}_i+{\beta}_9 RE{S}_i+\kern0.5em {\beta}_{10}{REG}_i++{\beta}_{11}{BTA}_I+{\beta}_{12}{FWT}_i+{\mu}_i $$

2 $$ \mathit{\ln}\left(\frac{\pi_i}{1-{\pi}_i\ }\right)={\beta}_0+{\beta}_1{NHIS}_i+{\beta}_2{WQ}_i+{\beta}_3{EDUC}_i+{\beta}_4{EMP}_i+{\beta}_5\ {MAR}_i+{\beta}_6{AGE}_i+{\beta}_7\kern0.50em {REL}_i+{\beta}_8{ETHN}_i+{\beta}_9 RE{S}_i+\kern0.5em {\beta}_{10}{REG}_i++{\beta}_{11}{BTA}_I+{\beta}_{12}{FWT}_i+{\mu}_i\kern11.75em $$

Where $\left(\frac{\lambda_i}{1-{\lambda}_i\ }\right)$ is the odds that a pregnant woman delivers in a health facility, and $\left(\frac{\pi_i}{1-{\pi}_i\ }\right)$ is the odds that the pregnant woman received a delivery care from a medical professional, *NHIS* represents the NHIS enrolment, *WQ* is the wealth quintile, *EDUC* is the level of education, *EMP* is the employment status, *MAR* is the marital status, *Age* denotes the age, *REL* is the religious affiliation, *ETHN*_*i*_ is the ethnicity variable, *RES* is the area of residence, *REG* represents the regional dummies, *BTA* denotes barriers to access and *FTV* is the frequency of watching television.

### Negative binomial estimation technique

The Negative Poisson estimation technique was used to analyse the third outcome variable “number of antenatal visits during pregnancy”. The choice of this estimation technique is underscored by the observation that the mentioned variable is not only a count variable, but preliminary diagnostic indicates that the variance exceeds the mean by 1.681. This cumulated into a problem of overdispersion which potentially biases the standard errors and the parameters of interest. The negative Binomial estimation technique is presented below:

3 $$ E(ANC)={\beta}_0+{\beta}_1{NHIS}_i+{\beta}_2{WQ}_i+{\beta}_3{EDUC}_i+{\beta}_4{EMP}_i+{\beta}_5\ {MAR}_i+{\beta}_6{AGE}_i+{\beta}_7\kern0.50em {REL}_i+{\beta}_8{ETHN}_i+{\beta}_9 RE{S}_i+\kern0.5em {\beta}_{10}{REG}_i++{\beta}_{11}{BTA}_I+{\beta}_{12}{FWT}_i+{\mu}_i $$

*E*(*ANC*) is the expected log count of the number of ANC visits, whereas the other covariates are in the case of Eqs. (1) and (2). Finally, the Incident Rate Ratio (IRR) was imposed on the expected log count of the number of ANC visits for the ease of interpretation and policy advocacy.

### Propensity score matching

This estimation technique enables the study to adjust for confounding effects and match women who are enrolled in the NHIS with those who are not enrolled. Given the binary nature of two of our dependent variables (place of delivery and assistance at delivery), we imposed a Linear Probability Model (LPM) assumption on their distributions to produce meaningful and intuitive corroborative results of the PSM. The PSM model for this study is stated as:

4 $$ {\pi}_i=E\left(\Delta  |{H}_i=1\right)=E\left({Y}_1|{X}_i,{H}_i=1\right)-E\left({Y}_0|{X}_i,{H}_i=1\right) $$

Where *Y*_1_ and *Y*_0_ are the potential outcomes (delivery care and ANC visits) corresponding to women who are enrolled in the NHIS and otherwise; *π*_*i*_ is the average treatment effect of a pregnant women enrolled in NHIS on delivery care and the number of ANC visits; *H* is the NHIS enrolment which is equal to 1; and *X* include women with similar propensities to be included in either the treated (enrolment) or the control group (non-enrolment) . The study adopted three main matching techniques, namely common support, nearest neighbour, and kernel in estimating Eq. (4). The bootstrap standard errors over 100 iterations were used to ensure robust results, whereas a seed of 1001 was used to guarantee the replicability of our PSM findings.

## Results

### Descriptive results

The sample characteristics provided in Table 1 indicate that the average number of ANC visits among pregnant women in Ghana is about six visits. Three out of four pregnant women make delivery in a health facility (75.6%). Similarly, three out of four pregnant women are assisted by a medical health practitioner during childbirth (76.4%). The table also indicates that the NHIS enrolment among pregnant women in the past five years preceding the 2014 GDHS survey is 66.8%. Majority of the women were rural dwellers (54.1%), were married (83.0%), were employed (82.7%) and were affiliated to the Christian religion (77.6%).

**Table 1:** Distribution of selected dependent and independent variables in Ghana

Variable	Obs	Mean	Std. dev	Min	max
Number of ANC visits	4169	6.474	2.876	0	20
Age	4169	30.632	7.025	15	49
Barriers to access	4169	−0.119	1.344	−1.187	4.013
**Variable**	**Obs**	**Frequency (%)**			
*Place of Delivery*					
Home	1015	24.3			
Health facility	3154	75.6			
*Birth attendant*					
Skilled birth attendant	985	23.6			
Unskilled birth	3184	76.4			
*Health Insurance (NHIS)*					
No	1384	33.2			
Yes	2785	66.8			
*Wealth Quintile*					
Poorest	873	20.9			
Poorer	847	20.3			
Middle	841	20.2			
Rich	1608	38.6			
*Education*					
No education	1068	25.6			
Primary	815	19.5			
At least secondary	2286	54.8			
*Employment*					
Not employed	721	17.3			
Employed	3448	82.7			
*Marital status*					
Never married	401	9.6			
Currently married	3462	83.0			
Formerly married	306	7.3			
*Religion*					
Christian	3234	77.6			
Moslem	652	15.6			
Traditional	122	2.9			
No religion	161	3.8			
*Ethnicity*					
Akan	2013	48.3			
Ga	272	6.5			
Ewe	565	13.6			
Northern	1319	31.6			
*Residence*					
Rural	2457	58.93			
Urban	1712	41.07			
*Region*					
Western	436	10.5			
Central	462	11.1			
Greater Accra	666	15.9			
Volta	315	7.6			
Eastern	397	9.5			
Ashanti	738	17.7			
Brong Ahafo	376	9.0			
Northern	489	11.7			
Upper East	177	4.2			
Upper West	113	2.7			
*Frequency of watching Television*					
Not at all	717	17.2			
At least once	3452	82.0			
Total	4169	100			

*Obs* number of observations

Source: Authors computation

### Bivariate logistics results for delivery care

In Table 2, we present the bivariate logistic regression results between the independent variables, place of delivery, and assistance at delivery across three samples-national, rural and urban sub-samples. The results indicate that, at the national level, women who are enrolled in the NHIS are 1.805 times more likely to deliver in a health facility compared to their counterparts who are not enrolled in the NHIS. This result is consistent across the rural sample (OR = 1.886, CI = 1.589–2.239) in terms of magnitude and significance. However, it was weakly significant at 10% level among the urban women (OR = 1.440*,* CI = 1.036–2.002). The same pattern is observed in terms of assisted delivery. However, the results for the urban sample was not significant. All the remaining control variables were intuitive and statistically significant.

**Table 2:** Bivariate logistics results for Delivery Care

	Place of Delivery			Assistance at Delivery		
	National	Rural	Urban	National	Rural	Urban
	OR	OR	OR	OR	OR	OR
NHIS (base: no)						
Yes	1.805^***^	1.886^***^	1.440^*^	1.864^***^	1.948^***^	1.498^*^
	(1.565–2.081)	(1.589–2.239)	(1.036–2.002)	(1.615–2.151)	(1.641–2.314)	(1.076–2.086)
Wealth Quintile (base: Poorest)						
Poorer	1.539^***^	1.537^***^	1.172	1.543^***^	1.560^***^	1.097
	(1.292–1.833)	(1.274–1.856)	(0.708–1.941)	(1.295–1.839)	(1.291–1.884)	(0.663–1.817)
Middle	3.061^***^	2.404^***^	2.356^***^	3.172^***^	2.562^***^	2.328^***^
	(2.505–3.739)	(1.889–3.061)	(1.493–3.718)	(2.589–3.886)	(2.005–3.275)	(1.471–3.685)
Richer	20.093^***^	9.554^***^	11.792^***^	22.783^***^	13.330^***^	12.480^***^
	(14.831–27.223)	(5.448–16.753)	(7.242–19.201)	(16.484–31.488)	(6.948–25.573)	(7.576–20.558)
Education (base: No education)						
Primary	1.876^***^	1.739^***^	1.916^**^	1.851^***^	1.723^***^	1.850^**^
	(1.566–2.248)	(1.412–2.142)	(1.272–2.887)	(1.544–2.219)	(1.398–2.124)	(1.221–2.801)
At least Secondary	5.477^***^	3.299^***^	7.584^***^	5.489^***^	3.398^***^	6.965^***^
	(4.630–6.480)	(2.709–4.018)	(5.190–11.082)	(4.631–6.507)	(2.784–4.148)	(4.765–10.182)
Employment status (base: Not employed)						
Employed	0.848	0.856	1.086	0.852	0.840	**1.166**
	(0.706–1.019)	(0.685–1.068)	(0.737–1.601)	(0.708–1.025)	(0.671–1.050)	(0.793–1.715)
Marital status (base: Never married)						
Currently married	0.610^***^	0.500^***^	1.252	0.587^***^	0.480^***^	1.200
	(0.465–0.802)	(0.357–0.699)	(0.760–2.062)	(0.444–0.775)	(0.341–0.676)	(0.721–1.996)
Formerly married	0.538^***^	0.448^***^	1.005	0.543^**^	0.453^***^	1.031
	(0.375–0.774)	(0.288–0.697)	(0.485–2.082)	(0.375–0.786)	(0.289–0.709)	(0.487–2.182)
Age	0.986^**^	0.982^***^	0.993	0.987^**^	0.981^***^	0.998
	(0.977–0.996)	(0.971–0.992)	(0.968–1.019)	(0.977–0.996)	(0.971–0.992)	(0.973–1.025)
Religion (base: Christian)						
Moslem	0.842	0.847	0.445^***^	0.851	0.858	0.451^***^
	(0.707–1.004)	(0.679–1.056)	(0.315–0.627)	(0.713–1.015)	(0.687–1.071)	(0.318–0.639)
Traditional	0.115^***^	0.206^***^	0.024^***^	0.115^***^	0.204^***^	0.024^***^
	(0.080–0.167)	(0.139–0.306)	(0.008–0.076)	(0.079–0.166)	(0.138–0.302)	(0.008–0.074)
No religion	0.250^***^	0.285^***^	0.245^***^	0.252^***^	0.291^***^	0.239^***^
	(0.182–0.344)	(0.195–0.417)	(0.116–0.515)	(0.184–0.346)	(0.199–0.424)	(0.114–0.504)
Ethnicity (base: Akan)						
Ga	1.002	0.726	1.508	0.868	0.630^*^	1.192
	(0.693–1.447)	(0.461–1.143)	(0.636–3.575)	(0.604–1.249)	(0.400–0.991)	(0.530–2.683)
Ewe	0.793	0.793	0.932	0.732^*^	0.746^*^	0.773
	(0.623–1.010)	(0.595–1.058)	(0.539–1.612)	(0.574–0.933)	(0.558–0.998)	(0.454–1.316)
Northern	0.475^***^	0.554^***^	0.533^***^	0.453^***^	0.522^***^	0.517^***^
	(0.408–0.554)	(0.461–0.664)	(0.379–0.751)	(0.388–0.529)	(0.434–0.628)	(0.364–0.733)
Residence (base: Urban)						
Rural	0.174^***^			0.178^***^		
	(0.146–0.208)			(0.149–0.213)		
Region (base: Greater Accra)						
Western	0.261^***^	0.821	0.398^*^	0.274^***^	0.834	0.434
	(0.168–0.407)	(0.447–1.510)	(0.170–0.930)	(0.174–0.431)	(0.449–1.548)	(0.183–1.029)
Central	0.239^***^	0.771	0.238^***^	0.260^***^	0.815	0.264^***^
	(0.154–0.372)	(0.418–1.421)	(0.110–0.513)	(0.166–0.407)	(0.437–1.518)	(0.121–0.576)
Volta	0.206^***^	0.713	0.187^***^	0.206^***^	0.698	0.187^***^
	(0.131–0.323)	(0.383–1.328)	(0.085–0.414)	(0.130–0.326)	(0.372–1.311)	(0.085–0.414)
Eastern	0.218^***^	0.624	0.361^*^	0.205^***^	0.585	0.291^**^
	(0.140–0.340)	(0.338–1.151)	(0.156–0.835)	(0.131–0.321)	(0.314–1.088)	(0.130–0.653)
Ashanti	0.617	1.445	0.615	0.635	1.428	0.666
	(0.380–1.002)	(0.743–2.809)	(0.268–1.412)	(0.388–1.041)	(0.726–2.809)	(0.286–1.547)
Brong Ahafo	0.348^***^	1.068	0.442	0.348^***^	1.037	0.442
	(0.223–0.544)	(0.579–1.971)	(0.194–1.006)	(0.221–0.547)	(0.556–1.931)	(0.194–1.006)
Northern	0.065^***^	0.190^***^	0.090^***^	0.065^***^	0.183^***^	0.092^***^
	(0.043–0.100)	(0.105–0.344)	(0.044–0.182)	(0.043–0.100)	(0.100–0.334)	(0.045–0.187)
Upper East	0.527^**^	2.034^*^	0.412^*^	0.536^*^	1.921^*^	0.502
	(0.329–0.844)	(1.078–3.839)	(0.173–0.978)	(0.332–0.867)	(1.008–3.659)	(0.204–1.236)
Upper West	0.199^***^	0.657	1.573	0.191^***^	0.605	1.573
	(0.127–0.310)	(0.358–1.205)	(0.338–7.325)	(0.121–0.300)	(0.327–1.119)	(0.338–7.325)
Barriers to Access	0.727^***^	0.769^***^	0.824^***^	0.721^***^	0.762^***^	0.812^***^
	(0.693–0.762)	(0.727–0.814)	(0.735–0.923)	(0.687–0.756)	(0.720–0.807)	(0.725–0.909)
Frequency of watching TV (base: Not all)						
At least one	2.344^***^	2.067^***^	2.364^***^	2.424^***^	2.146^***^	2.446^***^
	(1.994–2.755)	(1.704–2.506)	(1.636–3.414)	(2.061–2.851)	(1.769–2.603)	(1.691–3.538)
N	4169	2457	1712	4169	2457	1712
*R*^2^	0.013	0.016	0.004	0.015	0.018	0.005

*, **, and *** indicate 1%, 5%, and 10% levels of significance respectively. OR is Odds Ratio; Confidence Intervals in brackets; Coefficients are adjusted for clustering

Source: Authors computation

### Bivariate negative binomial regression results for the number of ANC visits

In Table 3, we present the incident rate of the bivariate negative binomial regression results of the association between the number of ANC visits and the independent variables. The results for the entire sample indicated that ANC visit was 1.114 times higher for women who were enrolled in NHIS compared to their counterparts who were not enrolled in the NHIS. Whereas the results for the rural women enrolled in the NHIS were significant with a higher incidence of ANC visits (IRR = 1.154, CI = 1.641–2.314) compared to the national sample, the results for the urban women who were enrolled are not statistically significant. The remaining control variables were intuitive and statistically significant.

**Table 3:** Bivariate negative binomial results for the number of ANC visits

	National	Rural	Urban
	IRR	IRR	IRR
NHIS (base: No)			
Yes	1.114^***^	1.154^***^	1.042
	(1.079–1.151)	(1.104–1.206)	(0.996–1.089)
Wealth Quintile (base: Poorest)			
Poorer	1.127^***^	1.141^***^	1.020
	(1.082–1.174)	(1.090–1.195)	(0.931–1.116)
Middle	1.234^***^	1.312^***^	1.044
	(1.187–1.283)	(1.249–1.378)	(0.970–1.124)
Richer	1.460^***^	1.477^***^	1.306^***^
	(1.414–1.508)	(1.395–1.565)	(1.224–1.393)
Education (base: No Education)			
Primary	1.126^***^	1.152^***^	1.038
	(1.080–1.173)	(1.094–1.212)	(0.968–1.111)
At least Secondary	1.284^***^	1.226^***^	1.213^***^
	(1.244–1.325)	(1.176–1.279)	(1.152–1.277)
Employment (base: Not employed)			
Employed	1.051^**^	1.040	1.085^**^
	(1.014–1.090)	(0.988–1.095)	(1.032–1.141)
Marital status (base: Never married)			
Currently married	1.050	1.006	1.121^***^
	(1.000–1.103)	(0.939–1.078)	(1.048–1.201)
Formerly married	1.019	0.993	1.068
	(0.946–1.098)	(0.897–1.100)	(0.959–1.190)
Age	1.003^***^	1.001	1.006^***^
	(1.001–1.005)	(0.998–1.004)	(1.003–1.009)
Religion (base: Christian)			
Moslem	0.958^**^	0.972	0.917^***^
	(0.928–0.990)	(0.928–1.018)	(0.878–0.958)
Traditional	0.656^***^	0.707^***^	0.614^***^
	(0.593–0.726)	(0.634–0.790)	(0.483–0.781)
No religion	0.739^***^	0.765^***^	0.757^**^
	(0.680–0.804)	(0.696–0.841)	(0.638–0.898)
Ethnicity (base: Akan)			
Ga	0.894^**^	0.793^***^	0.950
	(0.833–0.960)	(0.703–0.895)	(0.877–1.029)
Ewe	0.923^**^	0.923^*^	0.931^*^
	(0.879–0.968)	(0.861–0.989)	(0.873–0.994)
Guan	0.834^***^	0.825^***^	0.894^***^
	(0.810–0.858)	(0.792–0.858)	(0.859–0.931)
Residence (base: Urban)			
Rural	0.828^***^		
	(0.806–0.850)		
Regional (base: Greater Accra)			
Western	1.003	1.161	1.050
	(0.944–1.065)	(0.999–1.348)	(0.975–1.130)
Central	0.942	1.083	0.981
	(0.884–1.004)	(0.929–1.262)	(0.908–1.061)
Volta	0.809^***^	0.919	0.871^**^
	(0.751–0.872)	(0.782–1.080)	(0.790–0.960)
Eastern	0.756^***^	0.856^*^	0.808^***^
	(0.708–0.807)	(0.733–1.000)	(0.746–0.876)
Ashanti	0.988	1.178^*^	0.965
	(0.932–1.049)	(1.004–1.381)	(0.908–1.025)
Brong Ahafo	0.898^***^	1.039	0.926^*^
	(0.846–0.953)	(0.894–1.208)	(0.860–0.996)
Northern	0.655^***^	0.719^***^	0.777^***^
	(0.616–0.698)	(0.618–0.837)	(0.720–0.839)
Upper east	0.902^***^	1.040	0.954
	(0.852–0.954)	(0.898–1.204)	(0.886–1.027)
Upper west	0.780^***^	0.914	0.837^***^
	(0.736–0.827)	(0.789–1.059)	(0.774–0.906)
Barriers to Access	0.943^***^	0.945^***^	0.969^***^
	(0.933–0.953)	(0.932–0.958)	(0.953–0.986)
Frequency of watching TV (base: Not at all)	1.179^***^	1.206^***^	1.071^*^
At least one	(1.132–1.228)	(1.144–1.272)	
N	4169	2457	1712

*, **, and *** indicate 1%, 5%, and 10% levels of significance respectively. IRR is Incidence Rate Ratios ratio; Confidence Intervals in brackets; Coefficients are adjusted for clustering

Source: Authors construct

Women who were employed had a higher incidence of ANC visits (IRR = 1.051, CI =1.014–1.090) using the national sample. Although this finding was consistent across the urban sample, it was not the case across the rural sample. Additional increase in the age of women increased the occurrence of regular ANC visits in the national and urban samples. Results on the religious affiliation indicated that all other religions had a lower incidence of regular ANC visits compared to Christians at the national level. The same observation was made among the urban women, whereas for the rural sample, it was only consistent for the traditionalists and women with no religious affiliations. Women in all the ethnic groups had a lower incidence of regular ANC visits compared to the Akan women. The result on residence indicated that rural women had 0.828 lower incidence of regular ANC visits compared to their urban counterparts. The regional dummies mainly showed that women in other regions compared to the Greater Accra region had a lower frequency of ANC visits. Women who watch television at least once a week had a higher incidence of achieving several ANC visits compared to their counterparts who do not watch TV at all.

### Multivariate logistic regression results on effects of NHIS enrolment on delivery care

Table 4 presents the test for four hypotheses: (1) women who are enrolled in the NHIS are more likely to deliver in a health facility compared to their counterparts who are not enrolled; (2) rural women who are enrolled in the NHIS are more likely to deliver in a health facility compared to their urban counterparts who are enrolled; (3) women who are enrolled in the NHIS are more likely to use the services of a trained medical professional during childbirth compared to their counterparts who are not enrolled in the scheme; (4) rural women who are enrolled in the NHIS are more likely to use the services of a trained medical professional during childbirth compared to their urban counterparts who are not enrolled in the scheme.

**Table 4:** Multivariate Logistic Regression results of NHIS enrolment on Delivery Care

	Place of Delivery			Assistance at Delivery		
	National	Rural	Urban	National	Rural	Urban
	OR	OR	OR	OR	OR	OR
NHIS (base: No)						
Yes	1.697^***^	1.870^***^	1.145	1.819^***^	1.994^***^	1.263
	(1.425–2.022)	(1.533–2.281)	(0.774–1.695)	(1.524–2.171)	(1.631–2.438)	(0.847–1.881)
Wealth Quintile (base: Poorest)						
Poorer	1.403^**^	1.387^*^	1.329	1.382^**^	1.355^*^	1.311
	(1.115–1.764)	(1.076–1.788)	(0.731–2.417)	(1.098–1.740)	(1.049–1.750)	(0.720–2.386)
Middle	1.980^***^	1.881^***^	1.960^*^	2.054^***^	1.911^***^	2.141^*^
	(1.499–2.616)	(1.362–2.598)	(1.082–3.549)	(1.551–2.721)	(1.380–2.648)	(1.168–3.925)
Rich	7.650^***^	6.456^***^	6.957^***^	9.084^***^	8.482^***^	8.535^***^
	(5.078–11.525)	(3.528–11.815)	(3.477–13.919)	(5.934–13.906)	(4.239–16.972)	(4.230–17.223)
Education level (base: none)						
Primary	1.251	1.176	1.444	1.213	1.142	1.424
	(0.996–1.571)	(0.906–1.526)	(0.882–2.364)	(0.965–1.524)	(0.879–1.483)	(0.860–2.357)
At least Secondary	2.164^***^	1.831^***^	3.954^***^	2.065^***^	1.794^***^	3.362^***^
	(1.716–2.729)	(1.406–2.383)	(2.363–6.615)	(1.636–2.605)	(1.376–2.338)	(2.020–5.596)
Employment (base: Unemployed)						
Employed	1.105	1.113	1.214	1.112	1.093	1.298
	(0.883–1.383)	(0.858–1.444)	(0.768–1.919)	(0.884–1.397)	(0.839–1.424)	(0.821–2.052)
Marital status (base: currently married)	1.314	1.555^*^	0.769	1.419^*^	1.623^*^	0.942
Never married	(0.955–1.806)	(1.067–2.265)	(0.415–1.427)	(1.024–1.966)	(1.104–2.388)	(0.503–1.764)
Formerly married	0.928	0.919	0.945	0.990	0.979	1.030
	(0.684–1.259)	(0.649–1.300)	(0.473–1.886)	(0.728–1.347)	(0.692–1.385)	(0.503–2.110)
Age	1.000	0.998	1.002	1.001	0.999	1.008
	(0.988–1.012)	(0.985–1.012)	(0.976–1.029)	(0.989–1.013)	(0.985–1.012)	(0.981–1.035)
Religion (base: Christian)						
Moslem	1.139	1.178	0.986	1.184	1.222	0.952
	(0.897–1.448)	(0.897–1.547)	(0.563–1.726)	(0.930–1.506)	(0.929–1.608)	(0.544–1.668)
Traditional	0.308^***^	0.366^***^	0.058^***^	0.310^***^	0.373^***^	0.059^***^
	(0.202–0.468)	(0.235–0.570)	(0.020–0.172)	(0.203–0.471)	(0.239–0.581)	(0.021–0.162)
No religion	0.509^***^	0.491^***^	0.721	0.518^***^	0.507^***^	0.668
	(0.354–0.730)	(0.327–0.736)	(0.278–1.870)	(0.361–0.742)	(0.340–0.756)	(0.264–1.689)
Ethnicity (base: Akan)						
Ga/dangme	1.030	1.062	1.298	0.872	0.912	0.985
	(0.643–1.649)	(0.601–1.877)	(0.448–3.756)	(0.545–1.396)	(0.517–1.610)	(0.374–2.594)
Ewe	1.173	1.023	2.025	1.030	0.942	1.458
	(0.803–1.714)	(0.660–1.586)	(0.848–4.835)	(0.701–1.511)	(0.604–1.470)	(0.636–3.342)
Savannah	1.051	0.957	1.337	1.034	0.975	1.191
	(0.779–1.417)	(0.674–1.360)	(0.753–2.373)	(0.762–1.403)	(0.682–1.395)	(0.670–2.117)
Residence (base: urban)						
Rural	0.483^***^			0.532^***^		
	(0.382–0.612)			(0.420–0.674)		
Region (base: Greater Accra)						
western	0.703	1.076	0.552	0.687	0.988	0.543
	(0.409–1.208)	(0.503–2.302)	(0.207–1.475)	(0.396–1.191)	(0.458–2.128)	(0.200–1.475)
Central	0.754	1.199	0.485	0.790	1.186	0.522
	(0.442–1.284)	(0.558–2.575)	(0.204–1.155)	(0.458–1.360)	(0.545–2.578)	(0.217–1.252)
Volta	0.808	1.359	0.404	0.843	1.287	0.497
	(0.464–1.407)	(0.626–2.949)	(0.152–1.071)	(0.479–1.486)	(0.586–2.826)	(0.184–1.344)
Eastern	0.761	1.129	0.596	0.699	1.012	0.487
	(0.461–1.256)	(0.553–2.304)	(0.245–1.448)	(0.420–1.166)	(0.492–2.083)	(0.203–1.168)
Ashanti	1.361	2.287^*^	0.756	1.320	2.076	0.772
	(0.767–2.413)	(1.029–5.080)	(0.298–1.915)	(0.736–2.368)	(0.924–4.665)	(0.300–1.989)
Brong Ahafo	1.460	2.143	1.379	1.363	1.886	1.347
	(0.849–2.508)	(0.997–4.606)	(0.527–3.607)	(0.786–2.365)	(0.870–4.090)	(0.511–3.549)
Northern	0.507^*^	0.709	0.532	0.470^*^	0.597	0.588
	(0.288–0.893)	(0.316–1.590)	(0.206–1.376)	(0.263–0.838)	(0.264–1.352)	(0.222–1.555)
Upper East	4.106^***^	6.832^***^	1.827	3.915^***^	5.707^***^	2.455
	(2.212–7.619)	(2.958–15.780)	(0.605–5.516)	(2.089–7.336)	(2.445–13.323)	(0.786–7.662)
Upper west	1.408	1.912	3.527	1.224	1.527	3.602
	(0.781–2.537)	(0.845–4.325)	(0.713–17.437)	(0.672–2.230)	(0.669–3.485)	(0.723–17.949)
Barriers to Access	0.913^**^	0.900^**^	1.002	0.910^**^	0.897^**^	0.994
	(0.861–0.968)	(0.843–0.960)	(0.856–1.172)	(0.858–0.966)	(0.840–0.958)	(0.848–1.164)
Frequency of watching TV (base: Not at all)						
At least one	1.248^*^	1.260^*^	1.196	1.290^*^	1.300^*^	1.235
	(1.025–1.519)	(1.010–1.572)	(0.745–1.918)	(1.058–1.572)	(1.041–1.624)	(0.767–1.990)
N	4169.000	2457.000	1712.000	4169.000	2457.000	1712.000
R	0.250	0.160	0.230	0.255	0.167	0.234

*, **, and *** indicate 1%, 5%, and 10% levels of significance respectively; OR: Odds Ratio; Confidence Intervals in brackets; Coefficients are adjusted for clustering

Source: Authors computation

For the place of delivery, results at the national level indicated that women who were enrolled in the NHIS scheme were 1.697 times more likely to deliver in a health facility compared to their counterparts who were not enrolled. Women who were enrolled in the national sample, excluding residential effects, are 1.698 times more likely to deliver in a health facility compared to their counterparts who were not enrolled unto the scheme. The rural and urban sub-samples, however, recorded differential results of the effect of NHIS enrollment on the place of delivery. Whereas the rural sample indicated that women who were enrolled in the NHIS were 1.870 times more likely to deliver in a health facility with a statistical significance of 1%, the results for the urban sample was not statistically significant.

Concerning assistance at delivery, the results from the national level indicated that women who were enrolled in the NHIS scheme were 1.819 times more likely to be assisted by medical professionals during delivery compared to their counterparts who were not enrolled. Women who were enrolled in the national sample, excluding residential effects, are 1.821 times more likely to be assisted by a medical professional during delivery compared to their counterparts who were not enrolled unto the scheme. Whereas the rural sample indicates that women who are enrolled in the NHIS scheme are 1.994 times more likely to be assisted by a medical assistant during delivery with a statistical significance of 1%, the results for the urban sample was not statistically significant.

The results depict that woman of rich quintiles were more likely to deliver in a health facility (OR = 7.650; CI = 5.078–11.525) and to utilise the services of medical professionals (OR = 9.084; CI = 5.934–13.906) compared to their counterparts who are in the poorest wealth quintile. This pattern is consistent across the rural and urban samples, respectively. Women with at least secondary education were more likely to deliver in a health facility (OR = 2.164; CI = 1.716–2.729) and deploy the services of medical professionals (OR = 2.065; CI = 1.636–2.605) compared to women with no education. The results from the rural and urban samples are reflective of this pattern. Traditionalists and those without any religious affiliation were less likely to deliver in a health facility (OR = 0.308; CI = 0.202–0.468& OR = 0.509; CI = 0.235–0.570) and use the services of medical assistants (OR = 0.310; CI = 0.203–0.471& OR = 0.518; CI = 0.361–0.742) respectively. Rural women, in general, were less likely to deliver in a health facility (OR = 0.483; CI = 0.382–0.612) and use the services of medical professionals during childbirth (OR = 0.532; CI = 0.420–0.674).

### Multivariate negative binomial results of NHIS enrolment on the number of ANC visits

Table 5 provides the multivariate results for the effect of NHIS enrolment on the number of ANC visits using the entire sample and the rural-urban samples. The results from the Table are used to test two hypotheses: (1) women who are enrolled in the NHIS are more likely to make regular number of ANC visits compared to their counterparts who are not enrolled in the scheme; (2) rural women who are enrolled in the NHIS are more likely to make regular ANC visits compared to their urban counterparts who are not enrolled. The results from the national level indicated that women who are enrolled in the NHIS are 1.819 times more likely to make a regular number of ANC visits compared to their counterparts who are not enrolled. Women who are enrolled in the national sample, excluding residential effects, were 1.821 times more likely to make a regular number of ANC visits compared to their counterparts who are not enrolled unto the scheme.

**Table 5:** Multivariate Negative Binomial Regression results of effects of NHIS enrolment on the number of ANC visits

	National	Rural	Urban
	IRR	IRR	IRR
NHIS (base: No)			
Yes	1.099^***^	1.158^***^	1.023
	(1.066–1.134)	(1.110–1.208)	(0.981–1.066)
Wealth Quintile (base: Poorest)			
Poorer	1.059^*^	1.062^*^	1.035
	(1.013–1.107)	(1.008–1.120)	(0.945–1.133)
Middle	1.131^***^	1.193^***^	1.032
	(1.078–1.186)	(1.120–1.270)	(0.955–1.115)
Richer	1.273^***^	1.276^***^	1.226^***^
	(1.211–1.340)	(1.181–1.378)	(1.138–1.322)
Level of Education (base: No Education)			
Primary	1.037	1.045	1.015
	(0.994–1.081)	(0.990–1.103)	(0.946–1.088)
At least Secondary	1.082^***^	1.043	1.125^***^
	(1.041–1.125)	(0.991–1.098)	(1.058–1.196)
Employment (base: Unemployed)			
Employed	1.045^*^	1.042	1.046
	(1.009–1.083)	(0.991–1.096)	(0.996–1.100)
Marital Status (base: Currently married)	0.961	0.967	0.940
Never married	(0.915–1.010)	(0.900–1.039)	(0.878–1.006)
Formerly married	0.981	0.996	0.966
	(0.927–1.039)	(0.925–1.072)	(0.886–1.052)
Age	1.004^***^	1.003^*^	1.005^***^
	(1.002–1.006)	(1.000–1.005)	(1.002–1.008)
Religion (base: Christian)			
Moslem	1.052^**^	1.051	1.036
	(1.014–1.092)	(0.999–1.105)	(0.979–1.097)
Traditional	0.827^***^	0.850^**^	0.750^**^
	(0.752–0.910)	(0.767–0.943)	(0.603–0.932)
No religion	0.865^***^	0.880^**^	0.849
	(0.798–0.937)	(0.805–0.963)	(0.716–1.007)
Ethnicity (base: Akan)			
Ga/Dangme	0.948	0.952	0.946
	(0.886–1.014)	(0.842–1.077)	(0.873–1.024)
Ewe	1.035	1.100^*^	0.977
	(0.977–1.096)	(1.009–1.198)	(0.904–1.056)
Savannah	0.973	0.985	0.979
	(0.932–1.017)	(0.920–1.055)	(0.924–1.037)
Residence (base: urban)			
Rural	1.000		
	(0.969–1.033)		
Region (base: Greater Accra)			
Western	1.088^**^	1.192^*^	1.068
	(1.022–1.157)	(1.031–1.378)	(0.991–1.151)
Central	1.035	1.139	1.022
	(0.970–1.104)	(0.982–1.321)	(0.946–1.104)
Volta	0.916^*^	0.928	0.992
	(0.846–0.992)	(0.794–1.085)	(0.894–1.100)
Eastern	0.857^***^	0.933	0.857^***^
	(0.803–0.914)	(0.806–1.081)	(0.794–0.924)
Ashanti	1.037	1.233^**^	0.953
	(0.976–1.102)	(1.057–1.438)	(0.894–1.017)
Brong Ahafo	1.021	1.135	1.005
	(0.957–1.089)	(0.976–1.319)	(0.929–1.086)
Northern	0.849^***^	0.895	0.904^*^
	(0.787–0.915)	(0.760–1.055)	(0.824–0.992)
Upper east	1.120^**^	1.239^**^	1.079
	(1.043–1.203)	(1.054–1.456)	(0.990–1.177)
Upper west	0.955	1.067	0.889^*^
	(0.887–1.027)	(0.909–1.253)	(0.809–0.976)
Barriers to Access	0.987^*^	0.986^*^	0.995
	(0.977–0.998)	(0.973–0.999)	(0.980–1.011)
Frequency of watching TV (base: Not at all)			
At least one	1.033	1.070^**^	0.974
	(0.995–1.072)	(1.018–1.124)	(0.921–1.029)
N	4186.000	2468.000	1718.000
*R*^2^	0.043	0.041	0.033

*, **, and *** indicate 1, 5, and 10% levels of significance respectively. IRR: Incident Rate Ratio. Confidence intervals in brackets; Coefficients are adjusted for clustering

Source: Authors computation

The rural and urban sub-samples, however, recorded differential results of the effect of NHIS enrollment on the number of ANC visits. Whereas the rural sample indicates that women who are enrolled in the NHIS scheme are 1.994 times more likely to make regular ANC visits during pregnancy at a statistical significance of 1%, the results for the urban sample is not statistically significant. These variations in effects across the rural and urban samples depict the unevenness in the number of ANC visits across the country among women who are enrolled in the NHIS.

The wealth status of women revealed that regular ANC visits occurred among rich women compared to the poorest women (IRR = 1.273; *p* = 0.001). This finding was consistent across the rural and urban samples. Women with at least secondary education recorded higher occurrence of regular ANC visits compared to their counterparts who were not educated (IRR = 1.273; *p* = 0.001). This pattern was consistent across the rural and urban samples. The occurrence of regular ANC visits was 1.052 times higher among employed women compared to their counterparts who were not employed. However, this finding was only weakly significant and inconsistent across the sub-samples. The results depicted that ANC visit was lower among Traditionalist women (IRR = 0.827; *p* = 0.001) and those without any religious affiliation (IRR = 0.865; *p* = 0.001) compared to the Christians. The regional dummies indicated, compared to Greater Accra, that regular ANC visits occurred among women in the Western (IRR = 1.088; *p* = 0.05) and Upper East (IRR = 1.120; *p* = 0.05), whereas the reverse happened in the Eastern (IRR = 0.857; *p* = 0.001) and the Northern (IRR = 0.849. *p* = 0.001) regions.

### PSM results for the effects of NHIS enrolment on delivery care and ANC visits

The results for the PSM on Table 6 largely support the findings from the multivariate logistic and the negative binomial models on the effects of NHIS enrolment on the outcome variables across the entire and rural-urban sub-samples. Concerning Place of Delivery, the results from the PSM firm up the finding that enrolment in the NHIS increases Delivery in a health facility by 0.035 units among Ghanaian women in the national sample using the nearest neighbour matching technique, and that the decision to make a delivery in a health facility is increased by 0.103 units among rural women who are enrolled in the NHIS. However, this is not case for the urban women as none of the matching techniques recorded statistically significant results, though positive relationships were established.

**Table 6:** PSM results of NHIS enrolment on Delivery Care and ANC visits

	WomenEnrolled in NHIS (n^**T**^)	Women not Enrolled in NHIS (n^**C**^)	Average Effects	S.E.	Z Value
Place of Delivery					
*Common Support Matching Technique*					
National	2880	1289	0.035*	0.019	1.86
Rural	1651	806	0.103***	0.028	3.62
Urban	1219	483	0.010	0.020	0.5
*Nearest Neighbor Matching Technique*					
National	2880	1289	0.035***	0.016	2.16
Rural	1651	806	0.103***	0.029	3.61
Urban	1229	483	0.010	0.019	0.52
*Kernel Matching Technique*					
National	2880	1289	0.062***	0.017	3.58
Rural	1651	806	0.109***	0.022	4.93
Urban	1229	483	0.005	0.015	0.37
Assistance at Delivery					
*Common Support Matching Technique*					
National	2880	1289	0.045**	0.020	2.31
Rural	1651	806	0.027***	0.027	4.17
Urban	1229	483	0.015	0.021	0.7
*Nearest Neighbor Matching Technique*					
National	2880	1289	0.046**	0.021	2.13
Rural	1651	806	0.114***	0.029	3.9
Urban	1229	483	0.015	0.021	0.71
*Kernel Matching Technique*					
National	2880	1289	0.068***	0.016	4.33
Rural	1651	806	0.117***	0.022	5.22
Urban	1229	483	0.007	0.014	0.51
Number of ANC visits					
*Common Support Matching Technique*					
National	2880	1289	0.324**	0.124	2.62
Rural	1651	806	0.591***	0.152	3.89
Urban	1229	483	0.174	0.182	0.95
*Nearest Neighbor Matching Technique*					
National	2880	1289	0.330**	0.119	2.78
Rural	1651	806	0.591***	0.164	3.6
Urban	1229	483	0.187	0.181	1.03
*Kernel Matching Technique*					
National	2880	1289	0.432***	0.101	4.28
Rural	1651	806	0.626***	0.126	4.98
Urban	1229	483	0.233**	0.131	1.77

*, **, and *** indicate 1, 5, and 10% levels of significance respectively. n^T^: Treated Group, n^C^: Control Group

*S.E:* Bootstrap standard errors

Source: Authors computation

Concerning delivery assistance, the results from the PSM corroborate the finding that enrolment in the NHIS increases the usage of the services of medical assistants during delivery by 0.046 units using the nearest neighbour matching technique and that usage is also increased among rural women who are enrolled in the NHIS by 0.114 units using the mentioned matching technique. The case for the urban women was not statistically significant.

The outcomes from the PSM further concur the finding that enrolment in the NHIS increases regular number of ANC visits among Ghanaian women during pregnancy at the national level by 0.330 units using the nearest neighbour matching technique, and the effects on regular number of visits was highest among rural women (0.591 units) using the mentioned matching techniques. The case for the urban women, however, differed by not being statistically significant. This pattern was similar across all the matching techniques.

## Discussion

This study examined the rural-urban differences in the effect of NHIS enrolment on delivery care and ANC visits among Ghanaian women. The study produced four main findings: (1) statistical significant association between NHIS enrolment and delivery care among Ghanaian women at the national level; (2) rural women who are enrolled in the NHIS were more likely to utilise delivery care compared to their urban counterparts who were also enrolled in the NHIS; (3) statistical significant association between NHIS enrolment and ANC visits among Ghanaian women at the national level; and (4) rural women who are enrolled in the NHIS were more likely to make more ANC visits during pregnancy compared to their urban counterparts who were also enrolled in the NHIS.

The first and third findings indicate that women enrolled in the NHIS at the national level were more likely to access maternal health care (delivery care and ANC visits) compared to their counterparts who were not enrolled. These findings are consistent across the bivariate models, and even after controlling for demographic, socio-economic and locational factors in the multivariate models. Moreover, the findings are corroborated with available studies [13, 20]. The finding implies that NHIS enrolment will boost the demand for maternal health care utilisation among women beyond their peculiar characteristics and financial capability. Not surprisingly, [32, 33] found that direct financial barriers often mitigate maternal health care utilisation in developing countries. Premised on their findings, our results make an insightful contribution to the theory that enrolment in an NHIS among women in their reproductive years will increase their usage of maternal health services in Ghana by removing direct financial barriers [34], however, reported that health insurance coverage was associated with delivery but not ANC. Their study only focused on Northern and Central regions of Ghana, and this may account for the nuance in the findings.

Considering the second finding, the bivariate models of delivery care (place of delivery and assistance at delivery) using the entire sample, and the rural-urban sub-samples motivated the early evidence of inequalities among enrolment in NHIS and delivery care utilisation across the rural and urban women. This finding is striking given that when NHIS enrolment is controlled for in the entire sample using the multivariate models, rural women are less likely to utilise delivery care compared to their urban counterparts. However, the results from the rural and urban sub-samples revealed that rural women who are enrolled in the NHIS recorded a statistically significant association with the usage of delivery care, whereas the association between urban women who are enrolled, and the usage of delivery care was insignificant.

Comparison of the coefficients (in terms of the magnitude of the odds ratios) across the entire and the rural-urban subsamples for women enrolled in NHIS, revealed the coefficient of the rural-subsample as the highest. This implies that rural women who are enrolled are more likely to use delivery care services compared to their urban counterparts who are also enrolled across the country. The PSM results also corroborated this finding across all the three matching techniques that effects are higher and significant for women enrolled in the NHIS in rural areas compared to their urban counterparts who are also enrolled in the scheme. Our finding coincides with the observation made in western rural China after the introduction of the rural health insurance system [31].

The implications of this finding are that, without a social intervention like the NHIS, urban women are more likely to utilise delivery care compared to their rural counterparts, however, concentrating on both the rural and the urban samples exclusively vis-à-vis enrolment on the NHIS, rural women are more likely to utilise delivery care services compared to their urban counterparts. This positions the NHIS as an enabling factor in promoting universal access to maternal health care services in Ghana as stipulated by Goal 3.7 of the SDG [1], and as a pro-poor policy in bridging the within-country inequality in access to useful services such as delivery care and ANC utilisation. Observing that NHIS positively enhance healthcare utilisation among rural residents corroborated earlier findings [32, 33].

In terms of the fourth finding, the bivariate models of ANC using the entire sample, and the rural-urban sub-samples indicates early evidence of differences among enrolment in NHIS and number of ANC visits across the rural and urban samples. This finding suggests that when NHIS enrolment is controlled for in the entire sample using the multivariate models, rural women are less likely to make a higher number of ANC visits compared to their urban counterparts in general. However, the results from the rural and urban sub-samples revealed that rural women who are enrolled in the NHIS recorded a statistically significant association with the number of ANC visits, whereas the association between urban women who are enrolled, and the number of ANC visit is insignificant.

Comparing the coefficients (in terms of the magnitude of the incidence rate ratio) across the entire and the rural-urban subsamples among women enrolled in NHIS, revealed the coefficient of the rural-subsample as the highest. Meaning rural women who are enrolled are more likely to make a higher number of ANC visits compared to their counterparts who are also enrolled across the country. This pattern was supported by the PSM results across all the three matching techniques that, effects are higher and significant for women enrolled in the NHIS in the rural areas compared to their urban counterparts who are also enrolled in the scheme. This finding implies that, with a social intervention like the NHIS, rural women are more likely to make a regular number of ANC visits compared to their urban counterparts [34]. This finding makes the NHIS in Ghana a potential social equaliser in bridging the within-country inequality in access to maternal health service utilisation.

The overall implication of the four findings is that inequalities in the use and access of maternal health services among rural and urban women in their reproductive years can be bridged through an enabling factor as the NHIS. Also, given that financial barriers to health care access are pronounced among rural women, access and utilisation of antenatal and delivery care services will increase significantly. As a result, maternal mortality will reduce drastically, survival and well-being of both mother and her child enhanced. The results also suggest that problems such as anaemia and infections during pregnancy will be identified and treated even among pregnant women in the rural disadvantaged areas of the country. Ultimately, this will accelerate Ghana’s progress towards reducing maternal mortality deaths to less than 70 per 10,000 births before the year 2030 as prescribed by the SDG 3.1. Finally, our finding that NHIS potentially smoothens inequalities in health care utilisation between the rural and urban women contrasts the assertion that enabling factors exacerbate inequalities in healthcare utilisation [35].

It is worth noting that the binary logistic and negative binomial techniques used in the study do not permit causal inferences to be drawn between NHIS enrolment and maternal healthcare utilisation. These approaches at best could only establish association. However, the deployment of a quasi-experimental approach in thePSM may suggest some basis for a causal inference to be drawn for the national and the rural-urban effects. Even so, any assertion of causality necessitates further interrogation given the potential spill over among rural and urban women in the sample.

## Conclusion

Premised on the 2014 GDHS, which is a nationally representative data covering women between the ages of 15–49 across the rural and urban areas of Ghana, the rural-urban differences in the effects of enrolment in the NHIS on delivery care utilisation (place of delivery & assistance at delivery) and the number of ANC visits have been examined.

At the national level, the enrolment in NHIS has been observed to be correlated with delivery care utilisation and the number of ANC visits in Ghana. The use of PSM provides a basis to allude to the delivery care utilisation enhancing, and the number of ANC visits increasing effects of enrolment in NHIS among Ghanaian women. However, differences exist across the rural and urban women on the effects of enrolment in the NHIS on the utilisation of delivery care services and the number of ANC visits.

Whereas the rural sample indicated consistently that effects are more substantial in terms of the magnitude and significance across delivery care utilisation and the number of ANC visits, respectively, effects were invariably insignificant across the urban sample. These differences in outcomes in favour of the rural areas of the country which are mostly deprived and given that poverty is principally a rural phenomenon in Ghana, positions the NHIS as a potential social equaliser policy in health financing by increasing access to health care. The study recommends the NHIS as a social equaliser policy in health financing which should be increased among women in their reproductive lives with rural areas prioritised.

## Data Availability

The datasets generated during the current study are available on the Measure DHS website and can be directly assessed using https://dhsprogram.com/what-we-do/survey/survey-display-437.cfm.

## Abbreviations

ANC: Antenatal Care
CI: Confidence Interval
GDHS: Ghana Demographic Health Survey
GSS: Ghana Statistical Service
KMO: Kaiser-Meyer-Olkin
MMR: Maternal Mortality Rate
PSM: Propensity Score Matching
SDG: Sustainable Development Goal
WHO: World Health Organization

## Declarations

The Authors make the following declaration:

## References

[1] United Nations (2015). Transforming our world: the 2030 Agenda for Sustainable Development.

[2] Ghana Statistical Service (GSS), Ghana Health Service (GHS), ICF International (2015). Ghana demographic and health survey 2014.

[3] Ghana Statistical Service (GSS), Ghana Health Service (GHS), ICF (2018). Ghana Maternal Health Survey 2017.

[4] Afulani PA (2015). Rural/urban and socioeconomic differentials in quality of antenatal care in Ghana. PLoS One.

[5] Apanga PA, Awoonor-Williams JK (2018). Maternal death in rural Ghana: a case study in the upper east region of Ghana. Front Public Health.

[6] Government of Ghana (2003). National Health Insurance Policy Framework.

[7] World Health Organization (2018). Maternal Mortality.

[8] Alkema L, Chou D, Hogan D, Zhang S, Moller AB, Gemmill A, Fat DM, Boerma T, Temmerman M, Mathers C, Say L, United Nations Maternal Mortality Estimation Inter-Agency Group collaborators and technical advisory group (2016). Global, regional, and national levels and trends in maternal mortality between 1990 and 2015, with scenario-based projections to 2030: a systematic analysis by the UN maternal mortality estimation inter-agency group. Lancet.

[9] Abor P, Nkrumah G, Sakyi K, Adjasi C, Abor J (2011). The socio-economic determinants of maternal health care utilisation in Ghana. Int J Soc Econ.

[10] Adusi-Poku Y, Antwi E, Osei-Kwakye K, Tetteh C, Detoh E, Antwi P (2015). Quality of care: a review of maternal deaths in a regional Hospital in Ghana. Afr J Reprod Health / La Revue Africaine De La Santé Reproductive.

[11] Dzakpasu S, Soremekun S, Manu A, ten Asbroek G, Tawiah C, Hurt L, Fenty J, Owusu-Agyei S, Hill Z, Campbell OMR, Kirkwood BR (2012). Impact of free delivery care on health facility delivery and insurance coverage in Ghana’s Brong Ahafo region. PLoS One.

[12] Dixon J, Tenkorang EY, Luginaah IN, Kuuire VZ, Boateng GO (2014). National health insurance scheme enrolment and antenatal care among women in Ghana: is there any relationship?. Trop Med Int Health.

[13] Ghana Statistical Service (GSS) (2014). Ghana Living Standards Survey Round 6: Main Report. GSS, UNICEF, UNFPA, DFID.

[14] GSS (2008). Ghana Living Standards Survey Report of the fifth round (GLSS)*:* Accra: Ghana Statistical Service, 2005–06.

[15] Molini V, Paci P (2015). Poverty Reduction in Ghana—Progress and Challenges: Overview.

[16] Adomako J, Asare GQ, Ofosu A, Iott BE, Anthony T, Momoh AS, Warner EV, Idrovo JP, Ward R, Anderson FWJ (2016). Community-based surveillance of maternal deaths in rural Ghana. Bull World Health Organ.

[17] Boah M, Mahama AB, Ayamga EA (2018). They receive antenatal care in health facilities, yet do not deliver there: predictors of health facility delivery by women in rural Ghana. BMC Pregnancy Childbirth.

[18] Dickson KS, Adde KS, Amu H (2016). What influences where they give birth? Determinants of place of delivery among women in rural Ghana. Int J Reprod Med.

[19] Ameyaw EK, Kofinti RE, Appiah F (2017). National health insurance subscription and maternal healthcare utilisation across mothers’ wealth status in Ghana. Health Econ Rev.

[20] Adu-Gyamfi S, Brenya E, Adjei GN (2015). National health insurance and free maternal healthcare in Ghana: responses from women and health Workers in Akropong. Mod Res Stud.

[21] Twum P, Qi J, Aurelie KK, Xu L (2018). Effectiveness of a free maternal healthcare programme under the National Health Insurance Scheme on skilled care: evidence from a cross-sectional study in two districts in Ghana. BMJ Open.

[22] Bosomprah S, Ragno PL, Gros C, Banskota H (2015). Health insurance and maternal, newborn services utilisation and under-five mortality. Arch Public Health.

[23] Yaya S, Da F, Wang R, Tang S, Ghose B (2019). Maternal healthcare insurance ownership and service utilisation in Ghana: analysis of Ghana demographic and health survey. PLoS One.

[24] Novignon J, Ofori B, Tabiri KG, Pulok MH (2019). Socioeconomic inequalities in maternal health care utilisation in Ghana. Int J Equity Health..

[25] Wang W, Temsah G, Mallick L (2017). The impact of health insurance on maternal health care utilisation: evidence from Ghana, Indonesia and Rwanda. Health Policy Plan.

[26] Ameyaw EK, Dickson KS, Adde KS (2021). Are Ghanaian women meeting the WHO recommended maternal healthcare (MCH) utilisation? Evidence from a national survey. BMC Pregnancy Childbirth.

[27] Ameyaw EK, Ahinkorah BO, Baatiema L, Seidu AA (2021). Is the National Health Insurance Scheme helping pregnant women in accessing health services? Analysis of the 2014 Ghana demographic and health survey. BMC Pregnancy Childbirth.

[28] Sakeah E, Okawa S, Rexford Oduro A, Shibanuma A, Ansah E, Kikuchi K, Ghana EMBRACE Team (2017). Determinants of attending antenatal care at least four times in rural Ghana: analysis of a cross-sectional survey. Glob Health Action.

[29] Aday LA, Begley CE, Lairson DR, Slater CH (2004). Evaluating the healthcare system: effectiveness, efficiency, and equity.

[30] Jang Y, Chiriboga DA, Allen JY, Kwak J, Haley WE (2010). Willingness of older Korean-American adults to use hospice. J Am Geriatr Soc.

[31] Andersen RM, Yu H, Wyn R, Davidson PL, Brown ER, Teleki S (2002). Access to medical care for low-income persons: how do communities make a difference?. Med Care Res Rev.

[32] Tilahun H, Atnafu DD, Asrade G, Minyihun A, Alemu YM (2018). Factors for healthcare utilisation and effect of mutual health insurance on healthcare utilisation in rural communities of south Achefer Woreda, north west. Ethiop Health Econ Rev.

[33] Van Der Wielen N, Channon AA, Falkingham J (2018). Does insurance enrolment increase healthcare utilisation among rural-dwelling older adults? Evidence from the National Health Insurance Scheme in Ghana. BMJ Glob Health.

[34] Singh K, Osei-Akoto I, Otchere F, Sodzi-Tettey S, Barrington C, Huang C, Fordham C, Speizer I (2015). Ghana’s National Health insurance scheme and maternal and child health: a mixed methods study. BMC Health Serv Res.

[35] Addai I (2000). Determinants of use of maternal-child health services in rural Ghana. J Biosoc Sci.

